# DISRUPTING MECHANICAL HOMEOSTASIS PROMOTES MATRIX METALLOPROTEINASE-13 MEDIATED PROCESSING OF NEURON GLIAL ANTIGEN 2 IN MANDIBULAR CONDYLAR CARTILAGE

**DOI:** 10.22203/eCM.v045a08

**Published:** 2023-05-08

**Authors:** M. Bagheri Varzaneh, Y. Zhao, J. Rozynek, M. Han, D.A. Reed

**Affiliations:** 1 Department of Oral Biology, University of Illinois Chicago, Chicago IL, USA; 2 Department of Oral and Maxillofacial Surgery, University of Illinois Chicago, Chicago IL, USA

**Keywords:** Mandibular condylar cartilage, mechanobiology, cell-matrix interactions, protease, synovial fluid, temporomandibular joint, matrix metalloproteinase-13

## Abstract

Post-traumatic osteoarthritis in the temporomandibular joint (TMJ OA) is associated dysfunctional cell-matrix mediated signalling resulting from changes in the pericellular microenvironment after injury. Matrix metalloproteinase (MMP)-13 is a critical enzyme in biomineralisation and the progression of OA that can both degrade the extracellular matrix and modify extracellular receptors. This study focused on MMP-13 mediated changes in a transmembrane proteoglycan, Neuron Glial antigen 2 (NG2/CSPG4). NG2/CSPG4 is a receptor for type VI collagen and a known substrate for MMP-13. In healthy articular layer chondrocytes, NG2/CSPG4 is membrane bound but becomes internalised during TMJ OA. The objective of this study was to determine if MMP-13 contributed to the cleavage and internalisation of NG2/CSPG4 during mechanical loading and OA progression. Using preclinical and clinical samples, it was shown that MMP-13 was present in a spatiotemporally consistent pattern with NG2/CSPG4 internalisation during TMJ OA. *In vitro*, it was illustrated that inhibiting MMP-13 prevented retention of the NG2/CSPG4 ectodomain in the extracellular matrix. Inhibiting MMP-13 promoted the accumulation of membrane-associated NG2/CSPG4 but did not affect the formation of mechanical-loading dependent variant specific fragments of the ectodomain. MMP-13 mediated cleavage of NG2/CSPG4 is necessary to initiate clathrin-mediated internalisation of the NG2/CSPG4 intracellular domain following mechanical loading. This mechanically sensitive MMP-13-NG2/CSPG4 axis affected the expression of key mineralisation and OA genes including bone morphogenetic protein 2, and parathyroid hormone-related protein. Together, these findings implicated MMP-13 mediated cleavage of NG2/CSPG4 in the mechanical homeostasis of mandibular condylar cartilage during the progression of degenerative arthropathies such as OA.

## Introduction

TMJ dysfunction is a clinical syndrome affecting between 3–7 % of the population and can present with arthralgia, limited joint mobility, low-level inflammation, facial deformity, and a decreased quality of life. Degenerative arthropathies in the TMJ, including OA, constitute about 11 % of patients seeking treatment for TMJ disorders (Mejersjö and Hollender, 1984). While the aetiology of TMJ OA is unknown, a change in the mechanical homeostasis of the joint is a key initiating condition (Tanaka et al., 2008). Mechanical loading can impact cell-matrix interactions directly through the mechanical microenvironment of the pericellular matrix (Zelenski et al., 2015), and/or by potentiating the release of growth factors involved in the progression of OA (Chu et al., 2017; Vincent et al., 2004; Vincent et al., 2007; Vincent, 2013). These biochemical and biomechanical factors are dynamically regulated, in part, by a multitude of proteases that both degrade the matrix and affect cell signalling by modifying extracellular receptors (Guilak et al., 2018). In the TMJ, OA progression is associated with the activation of the serine protease HTRA1 that degrades pericellular type VI collagen, exposing the DDR2 receptor to type II collagen, and promoting the expression of MMP-13 (Polur et al., 2010; Xu et al., 2011; Xu et al., 2010). Prolonged exposure of the cartilage to activated MMP-13 compromises the integrity of the tissue due to proteolysis of type II collagen.

MMP-13 is critical for the progression of OA, with MMP-13 deficient mice displaying resistance to post-traumatic degradation of articular cartilage (Little et al., 2009; Wang et al., 2013). Targeting MMP-13 using broad-spectrum and MMP-13 specific inhibitors was not successful in clinical trials due to undesirable off-target effects (Renkiewicz et al., 2003; Whittaker et al., 2001; Xie et al., 2017). This finding underscored that MMP-13 functionality extends beyond matrix degradation, integrating cross talk between cell receptors, signalling networks, and growth factors. There is an important unmet need for characterising how MMP-13 can alter not only the matrix components of cartilage, but the extracellular receptors that interact with the pericellular microenvironment.

This study focused on NG2 (AN2 in mice; CSPG4 in humans; NG2/CSPG4). NG2/CSPG4 is a single pass, transmembrane proteoglycan that can bind with pericellular VI collagen (Burg et al., 1996) and is shed/cleaved by MMP-13 during anoikis (Joo et al., 2014). NG2/CSPG4 is present in a number of different cell types including polydendrocytes, macrophages, fibroblasts, pericytes, tenocytes, and chondrocytes. There are few mechanistic studies of NG2/CSPG4 in the musculoskeletal system. However, there is strong justification for the NG2/CSPG4 as an important regulators of cartilage health and disease because it binds with key OA growth factors such as FGF2 and PDGF (Goretzki et al., 1999) and is regulated by signalling pathways involved in cartilage homeostasis including ERK1/2 and PKC*α* (Makagiansar et al., 2007; Reed et al., 2022).

In cartilage, NG2/CSPG4 and its extracellular ligand type VI collagen are associated with both mineralisation and the progression of OA. In the epiphyseal growth plates of long bones during development, NG2/CSPG4 positive cells overlap with the presence of alkaline phosphatase and in hypertrophic cells. In cranial sutures, NG2/CSPG4 is expressed in the osteogenic bone front, implicating NG2/CSPG4 in both endochondral and intramembranous ossification (Fukushi et al., 2003). NG2/CSPG4 is further implicated in cartilage health and disease, expressed in human adult articular chondrocytes and losing the ability to bind with type VI collagen during OA (Midwood and Salter, 1998; Midwood and Salter, 2001). In type VI collagen knockout mice, biomineralisation of the TMJ and alveolar bone is impaired (Komori et al., 2022a; Komori et al., 2022b). In the TMJ, NG2/CSPG4 colocalises with pericellular type VI collagen in superficial/articular layer cells and is found within the cytosol in hypertrophic cells. NG2/CSPG4 translocates from the membrane to the cytosol in articular layer cells during the progression of TMJ OA (Yotsuya et al., 2019). While the mechanistic role of NG2/CSPG4 in cartilage has yet to be resolved, NG2/CSPG4 knockout mice have more severe cartilage degeneration during the early stages of TMJ OA and dysregulated mechanical activation of the ERK1/2 pathway (Reed et al., 2022).

The ability of NG2/CSPG4 to integrate inside-out and outside-in signalling requires complex processing steps by proteases, potentiating cell-signalling networks through regulated intramembrane proteolysis. This mechanism involves the proteolytic cleavage of the ectodomain followed by γ-secretase mediated release of the intracellular domain (Nayak et al., 2018; Sakry et al., 2014; Sakry and Trotter, 2016). The C-terminal, intracellular domain can impact transcriptional regulation of the cell through a PDZ binding motif (Barritt et al., 2000). Thus, post-translational modification to NG2/CSPG4 is an important determinant of functionality. Full length NG2/CSGP4 contains chondroitin sulphate chains and a 300 kDa core protein. Shedding or cleavage of the ectodomain releases either a 290 kDa fragment that contains the entire ectodomain or a 275/260 kDa fragment that lacks the intracellular domain and 64 amino acids from the N-terminal of the ectodomain (Asher et al., 2005; Nishiyama et al., 1995). This shed 275/260 kDa fragment is still tethered to the membrane due to non-covalent protein-protein interactions near the cleavage site. However, *in vivo* and *in vitro* studies have identified a range of fragment sizes from 130 to 275 kDa, suggesting the sequential processing after the initial cleavage event. This sequential processing is likely due to the bidirectional MMP-dependent interactions with MMP14, MMP-13, MMP9, and ADAM10 (Tamburini et al., 2019).

MMP14 cleavage is restricted to the distal most portion of the N-terminal between residues 490–500, likely removing the 64 base pair sequence at the N-terminal (Nishihara et al., 2015). MMP-13, MMP9, and ADAM10 are believed to cleave the ectodomain closer to the transmembrane portion of the protein in subdomain-3 (Asher et al., 2005; Nishiyama et al., 1995; Tamburini et al., 2019). Injury to the cerebral cortex is associated with an increase in shed NG2/CSPG4, with traumatic injury potentially releasing this diffusible, accumulated 290 kDa NG2/CSPG4 fragment. Shedding of the 290 kDa NG2/CSPG4 ectodomain fragment can be attenuated by broad-spectrum hydroxamic acid metalloproteinase inhibitors but not TIMPs. By contrast, generation of the 260/275 kDa ectodomain fragment is sensitive to both TIMP-2 and TIMP-3 (Asher et al., 2005). TIMP2 inhibits the gelatinase MMP2, while TIMP3 inhibits MMP-13 and ADAM10 (Ala-aho et al., 2002; Amour et al., 2000; Ni et al., 2021). MMP-13 is a known NG2/CSPG4 sheddase affecting during anoikis, with the accumulation of membrane-bound protein leading to the progression of apoptotic signalling (Joo et al., 2014). The 260/275 kDa shed, membrane-tethered fragment can be generated by blocking interactions of the NG2/CSPG4 ectodomain with the extracellular matrix using monoclonal antibodies (Nishiyama et al., 1995). During TMJ OA and following mechanical loading, there is a significant reduction in this shed, membrane-tethered NG2/CSPG4 fragment, indicating that MMP-13 may regulate this processing step of the NG2/CSPG4 ectodomain (Reed et al., 2022).

Taken together, these studies indicate that 1) MMP-13 and NG2/CSPG4 are abundant and associated with cartilage degeneration during OA progression, hypertrophy, and endochondral ossification, 2) that MMP-13 is a sheddase of the NG2/CSPG4 ectodomain, and that 3) NG2/CSPG4 is internalised in hypertrophic chondrocytes and articular fibrochondrocytes during TMJ OA. The role of mechanical loading and/or OA progression in generating select and specific NG2/CSPG4 fragment variants in an MMP-13 dependent manner, including internalisation of the intracellular domain, has yet to be fully resolved. The objective of this study was to determine if MMP-13 was present at the right time and place to be an NG2/CSPG4 sheddase during TMJ OA, to characterise the specificity of MMP-13 mediated activity on the NG2/CSPG4 ectodomain following mechanical loading, to determine if mechanical loading causes MMP-13 dependent internalisation of NG2/CSGP4, and to determine if this signalling axis regulates key OA and mineralisation pathways.

## Materials and Methods

### Control and NG2/CSPG4 knockout mice

Control mice from a C57 BL/6J background were purchased from Jackson Laboratory. Knockout mice were acquired from the KOMP repository (Cspg4tm1a(KOMP)Wtsi/Bcm, Davis, CA, USA). NG2/CSPG4 homozygous knockout mice are viable with a mild phenotype in the TMJ cartilage. The breeding diagram and TMJ phenotype for the NG2/CSPG4 knockout mice is reported in Reed et al. (2022). All animals were housed together to minimise confounding conditions. The use of all animal tissues followed an approved animal use protocol (UIC ACC #20-068).

### Preclinical surgical instability model of TMJ OA and synovial fluid collection

Post-traumatic TMJ OA was induced by unilateral partial discectomy according to the methods in Xu et al. (2009) and previous current authors’ publications (Reed et al., 2019; Reed et al., 2022; Reed et al., 2021; Yotsuya et al., 2019; Yotsuya et al., 2020). In short, 16-week-old c57 BL/6J mice were used for the study. This age of mouse represents skeletal maturity, and the experimental endpoint corresponds to a 38-year-old human, ensuring that observed degenerative changes are post-traumatic and not spontaneous in origin. For induction of post-traumatic TMJ OA, mice were anaesthetised with ketamine (100 mg/kg, Henry Schein, Dublin, OH, USA) and Xylazine (5 mg/kg, Akorn, Lake Forest, IL, USA), and the AD was excised on the right side of the animal through a 3–5 mm incision over the TMJ. Following excision, the joint was irrigated 1 × with sterile PBS. Sham control surgeries were identical except the disc remained intact. Tissues were collected at 2-, 4-, 8-, 12-, and 16-weeks after discectomy (diagram of the animal experiments is provided at Web ref. 1. Synovial fluid was collected following a published protocol (Øvlisen et al., 2009). In short, a 1 mm^2^ piece of sterile filter paper was inserted into the joint space until saturated with synovial fluid, with care to avoid contamination from blood. Filter paper was inserted into the joint space until no longer saturated. All filter paper samples were placed directly into 4 × Protein Sample Loading Buffer (928-40004, LI-COR, Lincoln, NE, USA), spun down on a desktop centrifuge to remove red blood cells, then the supernatant was heated for 10 min at 99 °C for denaturing, cooled to room temperature, and then placed in the −20 °C freezer for long term storage. A sample size of 4 was used for each experimental time point and determined by a sample size and power analysis prior to data collection. Each animal was considered an experimental unit. Exclusion criteria for the study included any animal that lost more than 20 % of their body weight or had a body condition score < 2. No animals met the exclusion criteria during the study. Experiments using vertebrate animals were approved by the UIC Animal Care Committee and performed in accordance with the relevant guidelines and regulations (UIC ACC #20-068).

### Clinical human samples of tissue and synovial fluid

Synovial fluid samples were collected intraoperatively from the clinical practice of the Department of Oral and Maxillofacial Surgery, UIC, from patients receiving arthrocentesis treatment for unilateral TMJ degeneration. Inclusion and exclusion criteria for the study followed previously published results (Reed et al., 2021). Synovial fluid samples were collected from the joint IL and CL to degeneration. The arthrocentesis procedure for both joints followed the standard of care for the procedure. The synovial fluid from each side was obtained at the beginning of the procedure, prior to the lysis and lavage portion. A 21-gauge inflow needle was introduced into the superior joint space, and after approximately 2–3 mL of injection of normal saline, the diluted synovial fluid was collected in a sterile tube in the operating suite, immediately placed on ice, and transported to the research laboratory. The sample was centrifuged to remove red blood cells and the supernatant was analysed for protein concentration using a NanoDrop 2000 Spectrophotometer (ND-2000, Thermo Fisher, Waltham, MA, USA). The sample was then mixed with 4 × Protein Sample Loading Buffer (928--40004, LI-COR), heated for 10 min at 99 °C for denaturing, cooled to room temperature, and then placed in the −20 ° freezer for long-term storage prior to western-blot analysis. Tissue samples used for immunohistochemistry were collected and processed as described in previously published work by the authors (Reed et al., 2022; Reed et al., 2021). In short, peri-articular tissue samples were collected intraoperatively during a total TMJ replacement surgery, placed in ice-cold 1 × PBS, and prepared for immunohistochemistry. All sample collection was approved by the institutional review board of UIC (IRB Protocol No. 2017-0033).

### Histomorphometry and immunohistochemistry of cartilage degeneration

TMJ tissue samples collected from control and TMJ OA mice were fixed in 4 % PFA overnight, decalcified with 4.5 % EDTA for 28 d, paraffin-wax embedded, and sectioned at 8 μm. Sections were stained using a Mason’s Trichrome. For immunohistochemistry, sections were dewaxed, treated with 132.2 mmol/L sodium borohydride, permeabilised with methanol and 0.5 % Triton (v/v), blocked in 5 % donkey serum (D9663, Sigma-Aldrich, St. Louis, MO, USA) for 2 h, and incubated with primary antibodies against either MMP-13 (1:200, AB39012, Abcam, Branford, CT, USA) or NG2/CSPG4 ectodomain (1:200, AB5320, Sigma-Millipore, Santa Cruz, CA, USA). All secondary labelling was with Alexa Fluor donkey anti-mouse 488 and donkey anti-rabbit 568 (1:500, Invitrogen, Invitrogen, Carlsbad, CA, USA). Nuclei were labelled with DAPI (D9542-1MG, 1 μg/μL, Sigma-Aldrich). Sections were imaged using an inverted fluorescent microscope using a 10 × objective (DMI6000B, Leica, Buffalo Grove, IL, USA). Laser intensity, gain, and magnification were standardised for all acquisitions. Brightness and contrast settings were standardised for all images during post-processing. Acquisition settings were verified using control stains containing no primary antibody or isotype control (Reed et al., 2022; Yotsuya et al., 2019). All images are representative of 4 biological replicates for each experimental group.

### Western-blot analysis

For protein isolation of monolayer-cultured cells, the culture medium was collected and placed on ice. The cells were washed 1 × in ice-cold PBS. For the NG2/CSPG4 western blot, cells were lysed in ice cold NP40 for 10 min (J60766.AP, Thermo Fisher, Waltham, MA, USA). For MMP-13 western blot, cells were lysed in M-PER (78501, Thermo Fisher). In both lysing protocols, the buffers include protease (cOmplete, 4693116001, Sigma-Aldrich) and phosphatase (PhosSTOP, 4906845001, Sigma-Aldrich) inhibitors. For protein isolation of cell culture media, media were concentrated using a centrifugal filter unit (Amicon Ultra-15, UFC901008, Sigma-Millipore) and mixed directly with the loading buffer. For *in vivo* tissues, tissue was homogenised in ice cold NP40 for 10 min (J60766.AP, Thermo Fisher). For *in vitro* protein isolation of the cell-agarose scaffolds, samples were rinsed in 1 × PBS for 20 min, placed in Laemmli Buffer (J60015.AC, Thermo Fisher), boiled for 10 min, cooled on ice, and spun down for 2 h using a mini-spin column to remove agarose (Pierce Spin Cups, 69700, Thermo Fisher). For all samples, lysate insolubles were removed by centrifugation at 14,000 ×*g* for 15 min at 4 °C. For monolayer cell and tissue samples tested for NG2/CSPG4, supernatant was incubated with Chondroitinase ABC (100330-1, AMSBio, Cambridge, MA, USA) added at 0.05 units/mL for 2 h at 37 °C. Lysates were adjusted to a 1 × Protein Sample Loading Buffer (928-40004, LI-COR), heated for 10 min at 99 °C for denaturing, run on a 4–15 % sodium dodecyl sulphate polyacrylamide gel (SDS-PAGE), and analysed by western blot with antibodies against the NG2/CSPG4 ectodomain (1:500, AB5320, Sigma-Millipore) or MMP-13 (1:500, AB39012, Abcam). All samples were fluorescently tagged with secondary antibodies (1:1000; IRDye 800CW Donkey anti-Rabbit IgG (H + L) or IRDye 680RD Donkey anti-Mouse IgG (H + L), LI-COR). Blots were imaged using a LI-COR Fluorescence Quantitative western blot. Fluorescence values were normalised to β-actin and standardised to experimental control samples. The western blot bands in the figures were cropped from blots that were imaged under identical acquisition settings. Changes in the brightness and contrast did not image the raw fluorescence reading from the bands. Full bands are provided at Web ref. 1, 2 and 3. *In vivo* samples from the MCC were collected from 4 biological replicates and 2 technical replicates. Synovial fluid samples were collected from 3 biological replicates. All *in vitro* samples were collected from 4 biological replicates.

### RT-qPCR

For quantifying gene expression changes from agarose samples, mRNA was isolated using the RNeasy Plant Mini Kit (74903, Qiagen, Germantown, MD, USA) due to the high levels of polysaccharides (Ogura et al., 2015). cDNA was generated from 500–1000 μg total RNA using a High-Capacity Reverse Transcription Kit (4368814, Applied Biosystems, Waltham, MA, USA). The lysate was then processed using the RNeasy Mini Kit (74104, Qiagen). All target genes were amplified with the SYBR^®^ Select Master Mix (4385610, Applied Biosystems) in a Bio-Rad iQ5 (Bio-Rad, Des Plaines, IL, USA). Primer sequences are reported in Table 1. All primers were validated using negative controls substituting molecular grade water for cDNA were carried out for each primer for standard quality control. Gene expression changes were calculated by comparative threshold cycle method with data standardised to a sample control and normalised to GAPDH using the ΔΔCq method. Negative controls with no cDNA were run for all primers. Four biological replicates and two technical replicates were used for each experimental group.

### Primary mandibular fibrochondrocyte isolation

The isolation of primary mandibular fibrochondrocytes followed published methods (Reed et al., 2022; Reed et al., 2021; Yotsuya et al., 2019), adapting the protocol for isolating primary chondrocytes (Bougault et al., 2009; Gosset et al., 2008). Primary cells from young mice were used because they maintain their chondrogenic phenotype for longer in cell culture conditions. In short, mandibular condyles from 10–14 day old control or NG2/CSPG4 knockout mice were excised by cutting the condylar process at the osteochondral transition zone. All muscle/ligament attachments were removed. The tissues were placed in CO_2_ independent collection medium (18045-088, Gibco, Gaithersburg, MD, USA) supplemented with 25 mg/mL Plasmocin (ant-mmp, InVivoGen, San Diego, CA, USA), 50 U/mL penicillin and 0.05 mg/mL streptomycin (P0781, Sigma-Aldrich), washed 1 × in sterile PBS, and transferred to a digestion DMEM (11966-025, Gibco) containing 3 mg/mL type II collagenase (S004174, Worthington Biochemical, Lakewood, NJ, USA) sterilised using a 0.2 μm filtered syringe. This digestion medium was replaced with a 1.5 mg/mL collagenase solution after 45 min and placed in an incubator with 5 % CO_2_ at 37 °C overnight. Cells were then dispersed by gentle agitation, with debris removed using a 40 μm cell strainer allowing the suspended cells of the MCC to pass through and the bone fragments to be retained in the strainer. Cells were isolated from the media with a 500 ×*g* centrifugation for 5 min at room temperature. The pellet was re-suspended in FBS supplemented advanced DMEM (12492-013, Gibco) and the cells were grown until confluent All experiments were carried out within the first 6 passages of the cells. MMP-13 inhibition experiments were carried out under serum starvation conditions, with cells cultured in Opti-MEM reduced serum media for no more than 24 h (31985, Gibco).

### Decellularisation

The decellularisation protocol follows published methods (Ravindran et al., 2012; Ravindran et al., 2015). In short, cells were grown to 100 % confluence, washed 1 × with PBS, and incubated in TBS containing 0.5 % triton X-100 (T8787-50ML, Sigma-Aldrich) for 1 h at 37 °C. The cells were then lysed with a 25 mmol/L solution of ammonium hydroxide for 20 min at 37 °C. Following lysis, the sample was again washed with TBS, treated with DNAse, and washed again. Decellularisation was validated using DAPI nuclear staining for all immunofluorescent imaging.

### In-cell/on-cell western-blot analysis

The in-cell/on-cell protocol was identical for cells and decellularised matrix. Samples were washed 1 × with PBS and then fixed overnight in Histochoice Tissue Fixative (VWRVH102, VWR, Radnor, PA, USA). For in-cell western blot, the cells were permeabilised using a 0.5 % triton X-100 for 15 min. For on-cell western blot, this step was conducted with 1 × PBS. Samples were then incubated in a LI-COR blocking buffer for 1 h at 37 °C. After blocking, cells were incubated overnight at 4 °C in a primary antibody mixed in LI-COR blocking buffer. Plates were immunolabelled using antibodies raised against either the NG2/CSPG4 intracellular domain (1:200, GGQPDPELLQFCRTPNPALRNGQYWV, UIC Protein Core, Chicago, IL, USA) or the NG2/CSPG4 ectodomain (1:200, AB5320, Sigma-Millipore). Samples were washed for 15 min in 0.5 % Triton X-100 at room temperature. All samples were fluorescently tagged with secondary antibodies (1:1000; IRDye 800CW Donkey anti-Rabbit IgG (H + L) or IRDye 680RD Donkey anti-Mouse IgG (H + L), LI-COR), washed 1 × in PBS, and imaged using LI-COR fluorescence quantitative western blot. Fluorescence values were collected from a region of interest containing only the cells. Raw fluorescence values were normalised to the untreated controls. All quantified data represents four biological replicates.

### Cell-agarose-collagen scaffolds and inhibition of MMP-13

Primary mandibular condylar fibrochondrocytes were seeded in agarose-collagen scaffolds and loaded in a compression bioreactor as previously described (Reed et al., 2021). Cells were isolated in 1 mL of supplemented advanced DMEM (12492-013, Gibco) and mixed with a 5 % low-gelling temperature agarose and 0.25 mg/mL rat tail collagen (A1048301, Thermo Fisher) when the solution reached 45 °C, generating a 4 % agarose/collagen solution at a density of 4 × 10^5^ cells/mm^3^ (Reed et al., 2022). The scaffolds were cast by pouring the aqueous solution on a 1 mm thick glass spacer plate (1653311, Bio-Rad), yielding a cell-agarose-collagen scaffold of uniform 1 mm thickness. After 30 min, a punch was used to generate plugs measuring 17 mm in radius. The cell-agarose/collagen scaffold plugs were immediately placed in supplemented advanced DMEM (12492-013, Gibco) and cultured for 72 h at 37 °C and 5 % CO_2_ to allow the cells time to generate endogenous pericellular type VI collagen. For mechanical loading, cell-agarose/collagen scaffolds were placed in a compression bioreactor housed inside a cell incubator at 37 °C and 5 % CO_2_ and loaded under constrained, uniaxial compression at 2.5 N for 2 h. Samples were pre-treated with either a vehicle control (DMSO, 1 μL, Sigma-Aldrich) or inhibitor of MMP-13 (CAS 544678-85-5, 1 mmol/L, 1 μL, Calbiochem, San Diego, CA, USA) for 24 h under serum free conditions. The concentration and duration of the MMP-13 treatment was chosen to replicate published results (Joo et al., 2014). Four biological replicates were used for each experimental group.

### Immunocytochemistry and colocalisation analysis

Cell-agarose-collagen scaffolds were fixed overnight in Histochoice tissue fixative (VWRVH102, VWR). Samples were cryo-embedded using OCT compound and cryo-sectioned at 20 μm (CM1950, Leica). Slides were immunolabelled, following the immunohistochemistry protocol previously described, with primary antibodies raised against the NG2/CSPG4 intracellular domain (1:200, GGQPDPELLQFCRTPNPALRNGQYWV, UIC Protein Core), NG2/CSPG4 ectodomain (1:200, AB5320, Sigma-Millipore), MMP-13 (1:200, AB39012, Abcam) and/or CHC (1:200, MA1-065, Thermo Fisher). Images were acquired using a laser scanning confocal microscope with 63 × oil immersion objective (LSM 710, Zeiss, White Plains, NY, USA). The laser intensity, brightness, and gain were standardised for all image acquisitions. Colocalisation coefficients and fluorescence/channel intensity were calculated using ImageJ (Rasband, 2011) using the Pearson’s overlap coefficient were compare (Adler and Parmryd, 2010) and the Costes method. Images were quantified by personnel blind to the study design, outcome assessment, and analysis. Four biological replicates and 5 technical replicates for each biological replicate was used for each experimental group.

### Statistical analysis

A one-way ANOVA was used for all statistical tests. *Post hoc* Bonferroni tests were carried out for multigroup comparisons (SPSS, Chicago, IL). Bonferroni tables are provided at Web ref. 4. A *p*-value < 0.05 was considered statistically significant for all studies.

## Results

### The loss of NG2/CSPG4 and increase in MMP-13 is temporally correlated in early stage TMJ OA

**Fig. 1.**
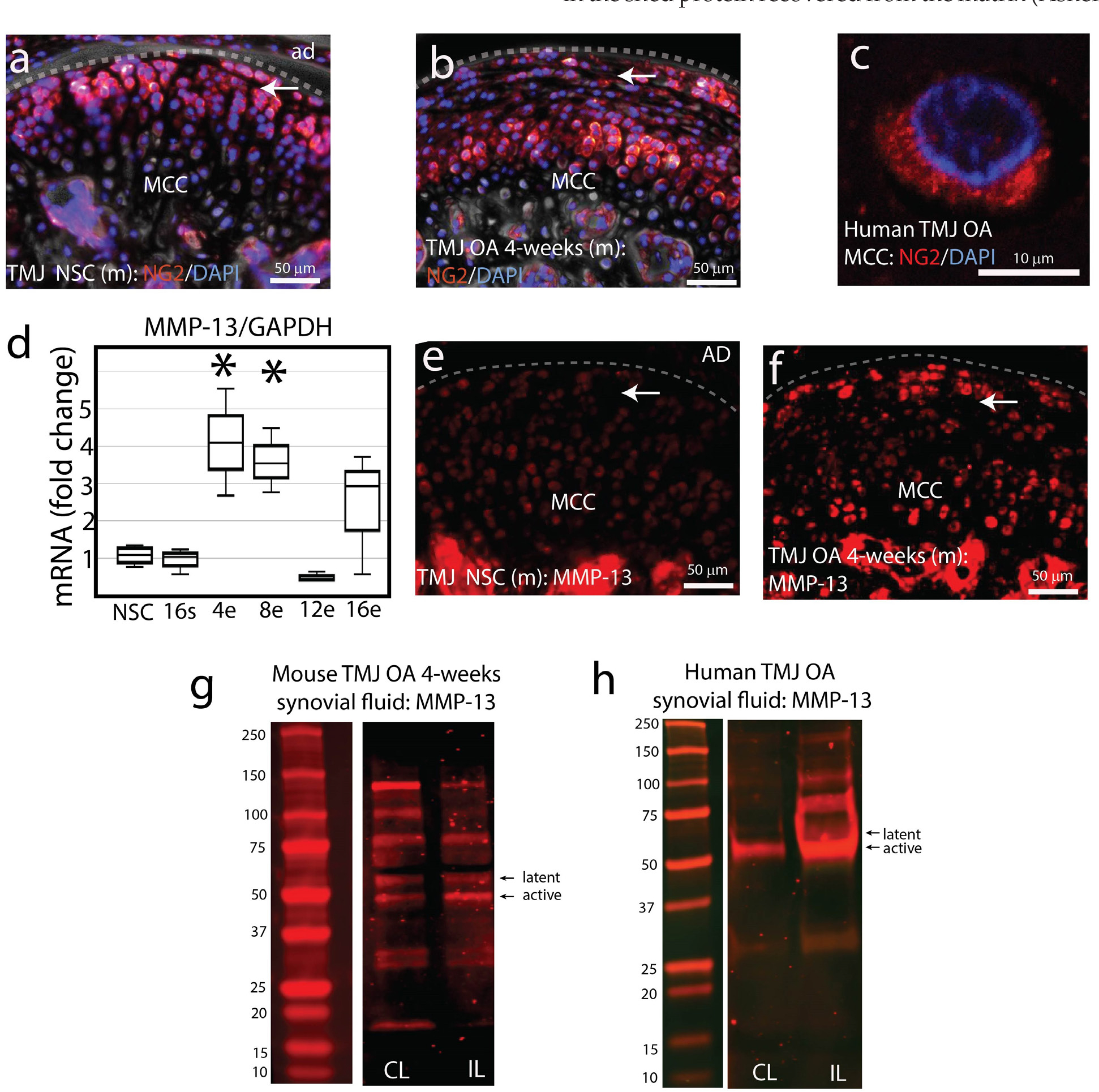
Loss of NG2/CSPG4 is associated with elevated MMP-13 in early stage TMJ OA. (**a**) Immunohistochemistry of NG2/CSPG4 protein in mouse (m) NSC TMJ tissue with the MCC and AD. Dashed line indicates the articular surface of the MCC. Arrow indicates the superficial/articular layer cells. (**b**) Immunohistochemistry of NG2/CSPG4 protein in mouse 4-week TMJ OA tissue. Dashed line indicates the articular surface of the MCC. Arrow indicates the superficial/articular layer cells. (**c**) Immunohistochemistry of the NG2/CSPG4 intracellular domain protein from human TMJ OA MCC. (**d**) RT-qPCR analysis of mandibular condyles collected at each stage of TMJ OA illustrating a significant increase in MMP-13 gene expression at 4- and 8-weeks post-injury relative to the NSCs. Data standardised and normalised to GAPDH using the ΔΔCq. (**e**) Immunohistochemistry of MMP-13 protein in mouse NSC TMJ tissue. Dashed line indicates the articular surface of the MCC. Arrow indicates the superficial/articular layer cells. (**f**) Immunohistochemistry of MMP-13 protein in mouse 4-week TMJ OA tissue. Dashed line indicates the articular surface of the MCC. Arrow indicates the superficial/articular layer cells. (**g**) Western-blot analysis of MMP-13 protein levels in a 4-week TMJ OA mouse from synovial fluid samples collected CL and IL to injury. (**h**) Western-blot analysis of MMP-13 protein levels in a unilateral human TMJ OA case from synovial fluid samples collected during arthrocentesis CL and IL to injury. * = *p* < 0.05 for comparisons relative to NSC samples. *n* = 4/experimental group for RT-qPCR quantification.

To characterise changes in NG2/CSPG4 during the progression of TMJ OA, immunohistochemistry was carried out on mouse control and OA TMJ tissues using a polyclonal antibody raised against the NG2/CSPG4 ectodomain (AA 30-2225) at 4-weeks after the discectomy. Compared to the NSC samples, superficial and chondroblastic cells in the TMJ OA sample had lower levels of NG2/CSPG4 (Fig. 1a,b). These superficial layer TMJ OA cells had elevated levels of internalised NG2/CSPG4 using a monoclonal antibody raised against the intracellular domain (AA 2291–2322), illustrated here in an articular fibrochondrocyte from a human TMJ OA condyle extracted during a total joint replacement procedure (Fig. 1c). To determine if MMP-13 was present in superficial/articular layer cells in a spatiotemporally-correlated pattern with this observed reduction in NG2/CSPG4, RT-qPCR analysis was conducted on control and OA MCC and showed significantly elevated levels of MMP-13 expression at 4- and 8-weeks following discectomy when compared to the controls (Fig. 1d). To determine if MMP-13 was spatially associated with NG2/CSGP4 positive cells, immunohistochemistry was conducted on paraffin-wax-embedded tissues at 4-weeks post-discectomy. Data illustrate that the highly cellular neo-matrix forming over the top of the articular layer of MCC contains a high number MMP-13-positive cells when compared to the non-surgical tissues that contained no MMP-13 immunopositive cells in the articular/superficial layer (Fig. 1e,f). Since MMP-13 can be secreted from a variety of cells including synoviocytes, synovial fluid samples from the preclinical mouse model were screened using western-blot analysis. Distinct latent and active MMP-13 bands were observed 4-weeks after discectomy in the synovial fluid both contra- and IL to discectomy (Fig. 1g), compared with non-surgical and sham control samples (Supporting data provided at Web ref. 1). This finding was validated translationally with a clinical human synovial fluid sample collected from a patient with unilateral TMJ cartilage degeneration (Fig. 1h). In both the preclinical animal model sample and the clinical human sample, 2 distinct bands were observed, illustrating that MMP-13 is present in both the latent and active forms in the synovial fluid.

### Inhibition of MMP-13 prevents retention of NG2/CSPG4 in the extracellular matrix

**Fig. 2.**
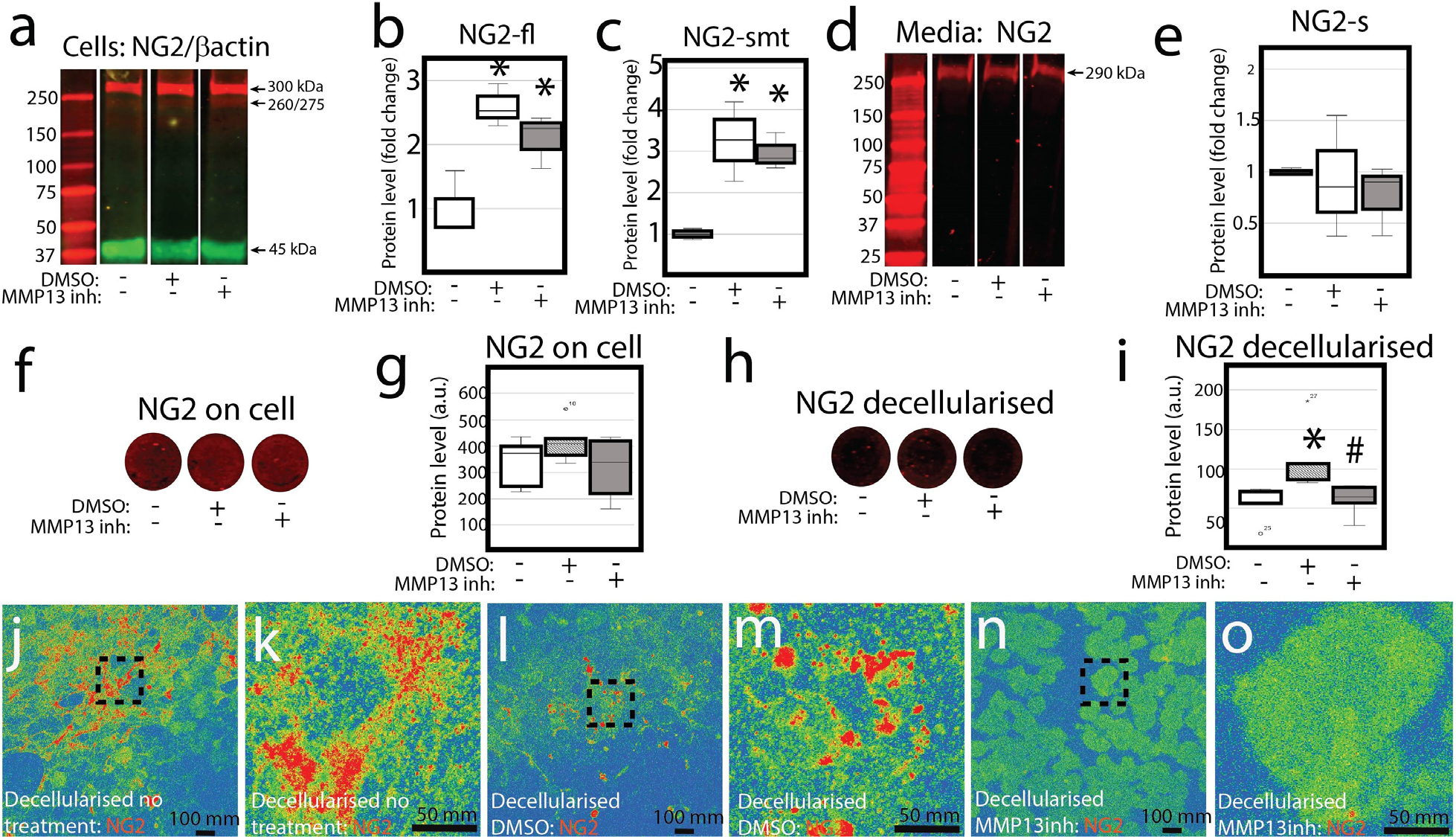
NG2/CSPG4 is a substrate for MMP-13 mediate binding with the extracellular matrix. (**a**) Western-blot analysis of NG2/CSPG4 protein in primary mandibular fibrochondrocyte cells treated with either DMSO or MMP13inh. (**b**) Quantification of the NG2/CSPG4 full length band (300 kDa; NG2-fl). (**c**) Quantification of the NG2/CSPG4 shed, membrane tethered band (260/275 kDa; NG2-smt). (**d**) Western blot analysis of shed NG2/CSPG4 protein (NG2-s) from the cell culture media of primary mandibular fibrochondrocytes treated with either DMSO or MMP13inh. (**e**) Quantification of the NG2/CSPG4 shed fragment (290 kDa; NG2-s). (**f**) On-cell western blot of total NG2/CSPG4 protein levels from primary mandibular fibrochondrocyte cells treated with either DMSO or MMP13inh. (**g**) Quantification of the total NG2/CSPG4 protein levels from the on-cell western blot. (**h**) On-cell western blot of total NG2/CSPG4 protein levels from a decellularised extracellular matrix from primary mandibular fibrochondrocyte cells treated with either DMSO or MMP13inh. (**i**) Quantification of the total NG2/CSPG4 protein levels from the on-cell western blot from a decellularised extracellular matrix. (**j**,**k**) Immunocytochemistry of NG2/CSPG4 from the decellularised extracellular matrix from untreated primary mandibular fibrochondrocyte pseudo coloured to illustrate fluorescence intensity. (**l**,**m**) Immunocytochemistry of NG2/CSPG4 from the decellularised extracellular matrix from primary mandibular fibrochondrocyte treated with DMSO pseudo coloured to illustrate fluorescence intensity. (**n**,**o**) Immunocytochemistry of NG2/CSPG4 from the decellularised extracellular matrix from primary mandibular fibrochondrocyte treated with an MMP13inh pseudo coloured to illustrate fluorescence intensity. * = *p* <0.05 for group comparisons between unloaded and loaded samples. # = *p* < 0.05 for group comparisons between DMSO and MMP-13 inhibited samples. *n* = 3/experimental group for western blot quantification. *n* = 5/experimental group for on cell western blot quantification.

Studies in other cell types have illustrated that proteolytic shedding of NG2/CSPG4 leads to a reduction in the full-length protein and an increase in the shed protein recovered from the matrix (Asher et al., 2005; Joo et al., 2014). To determine if MMP-13 has a similar proteolytic role on NG2/CSPG4 in mandibular fibrochondrocytes, primary mandibular fibrochondrocytes were cultured in monolayer with and without a small molecule inhibitor of MMP-13. The addition of DMSO elevated the amount of total and shed, membrane tethered NG2/CSPG4. Compared to the DMSO control, inhibiting MMP-13 had no significant impact on the amount of full length or shed, membrane tethered NG2/CSPG4 (Fig. 2a,b,c); nor did it influence the amount of shed NG2/CSPG4 recovered from the media (Fig. 2d,e). Since NG2/CSPG4 binds to type VI collagen and is implicated in regulating anoikis (Joo et al., 2008; Joo et al., 2014), whether MMP-13 affects the retention of NG2/CSPG4 in the matrix was tested next. Protein levels from cells quantified by on-cell western blot suggested no effect of MMP-13 (Fig. 2f,g). Repeating the experiment in a decellularised matrix illustrated that inhibiting MMP-13 reduced the retention of the NG2/CSPG4 ectodomain in the extracellular matrix when compared with DMSO (Fig. 2h,i). To replicate the finding, immunocytochemistry was performed on a decellularised matrix, immunolabeling with a polyclonal antibody raised against the NG2/CSPG4 ectodomain. The inhibition of MMP-13 led to a dramatic reduction of immunopositive NG2/CSPG4 retained in the matrix when compared to the non-treatment and DMSO controls (Fig. 2j–o).

### MMP-13 regulated the accumulation of membrane bound NG2/CSPG4 but not mechanical loading mediated degradation of the ectodomain

**Fig. 3.**
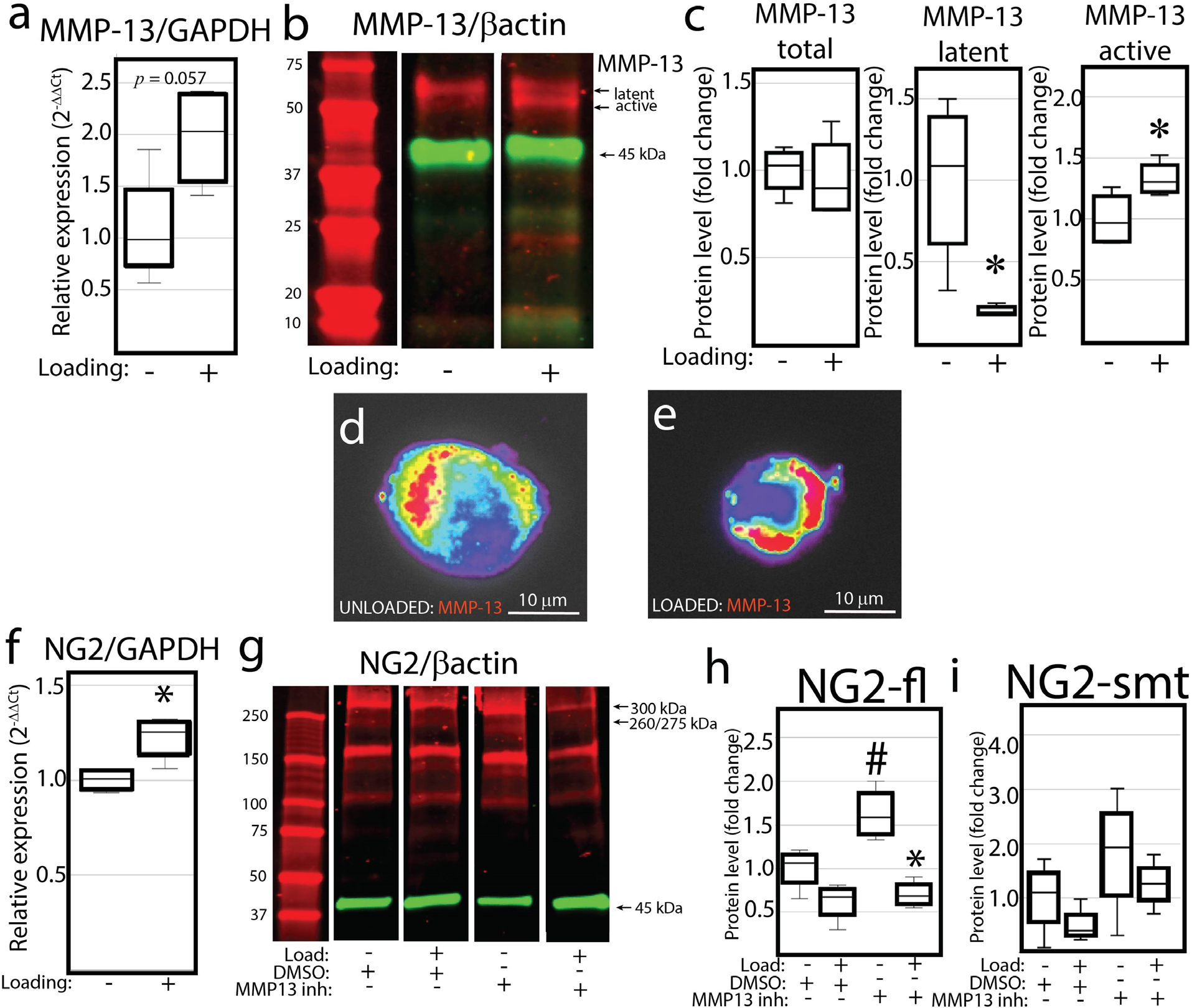
Mechanical loading activates MMP-13 but does not degrade the NG2/CSPG4 ectodomain. (**a**) RT-qPCR of MMP-13 in unloaded and loaded primary mandibular fibrochondrocytes. Data standardised to the unloaded control and normalised to GAPDH using the ΔΔCq method. (**b**) Western-blot analysis of MMP-13 in unloaded and loaded primary mandibular fibrochondrocytes. The two MMP-13 bands correspond to a latent 60 kDa fragment and an active 48 kDa fragment. (**c**) Quantification of the western blot for the total, latent, and active MMP-13. (**d**,**e**) immunocytochemistry of MMP-13 in unloaded and loaded primary mandibular fibrochondrocytes with a gradient pseudo colour to illustrate fluorescence intensity. (**f**) RT-qPCR of NG2/CSPG4 in unloaded and loaded primary mandibular fibrochondrocytes. Data standardised to the unloaded control and normalised to GAPDH using the ΔΔCq method. (**g**) Western-blot analysis of NG2/CSPG4 in unloaded and loaded primary mandibular fibrochondrocytes treated with either DMSO or an MMP13inh. (**h**) Quantification of the NG2/CSPG4 full length band (300 kDa; NG2-fl). (**i**) Quantification of the NG2/CSPG4 shed, membrane tethered band (260/275 kDa; NG2-smt). All data are standardised to the unloaded vehicle control and normalised to βactin. * = *p* < 0.05 for group comparisons between unloaded and loaded samples. # = *p* < 0.05 for group comparisons between DMSO and MMP-13 inhibited samples. *n* = 4/experimental group for western blot and RT-qPCR quantification.

To determine if mechanical loading altered MMP-13 proteolytic activity, mandibular fibrochondrocytes embedded in a 4 % agarose-collagen scaffold were loaded at 2.5 N for 2 h. RT-qPCR analysis showed that mechanical loading increased the expression of MMP-13, but not significantly (Fig. 3a). Western-blot analysis showed that constrained static compression did not alter total MMP-13 protein but did promote a significant shift from the latent 60 kDa fragment to the active 48 kDa fragment (Fig. 3b,c). The separation of the two MMP-13 fragments was confirmed using a plot profile (Supporting data provided at Web ref. 3). Immunocytochemistry of the loaded and unloaded cells showed that MMP-13 was localised to the pericellular microenvironment around the cell, indicating that it spatially overlaps with the NG2/CSPG4 ectodomain (Fig. 3d,e). For NG2/CSPG4, RT-qPCR analysis showed that mechanical loading significantly increased the expression of NG2/CSPG4 (Fig. 3f). Culturing the three-dimensional scaffolds with an MMP13inh led to the accumulation of membrane bound, full-length NG2/CSPG4. Inhibition of MMP-13 protein was confirmed by western blot (Supporting data provided at Web ref. 3). Mechanical loading resulted in a reduction of both the full-length and shed membrane-tethered fragments of NG2/CSPG4 in both the DMSO and MMP-13 inhibited samples (Fig. 3g–i). Together, these data illustrated that MMP-13 regulated the accumulation of membrane bound NG2/CSPG4 but did not affect the formation of mechanical-loading-dependent variant-specific fragments of the ectodomain.

### MMP-13 regulates clathrin-mediated internalisation of the NG2/CSPG4 intracellular domain

**Fig. 4:**
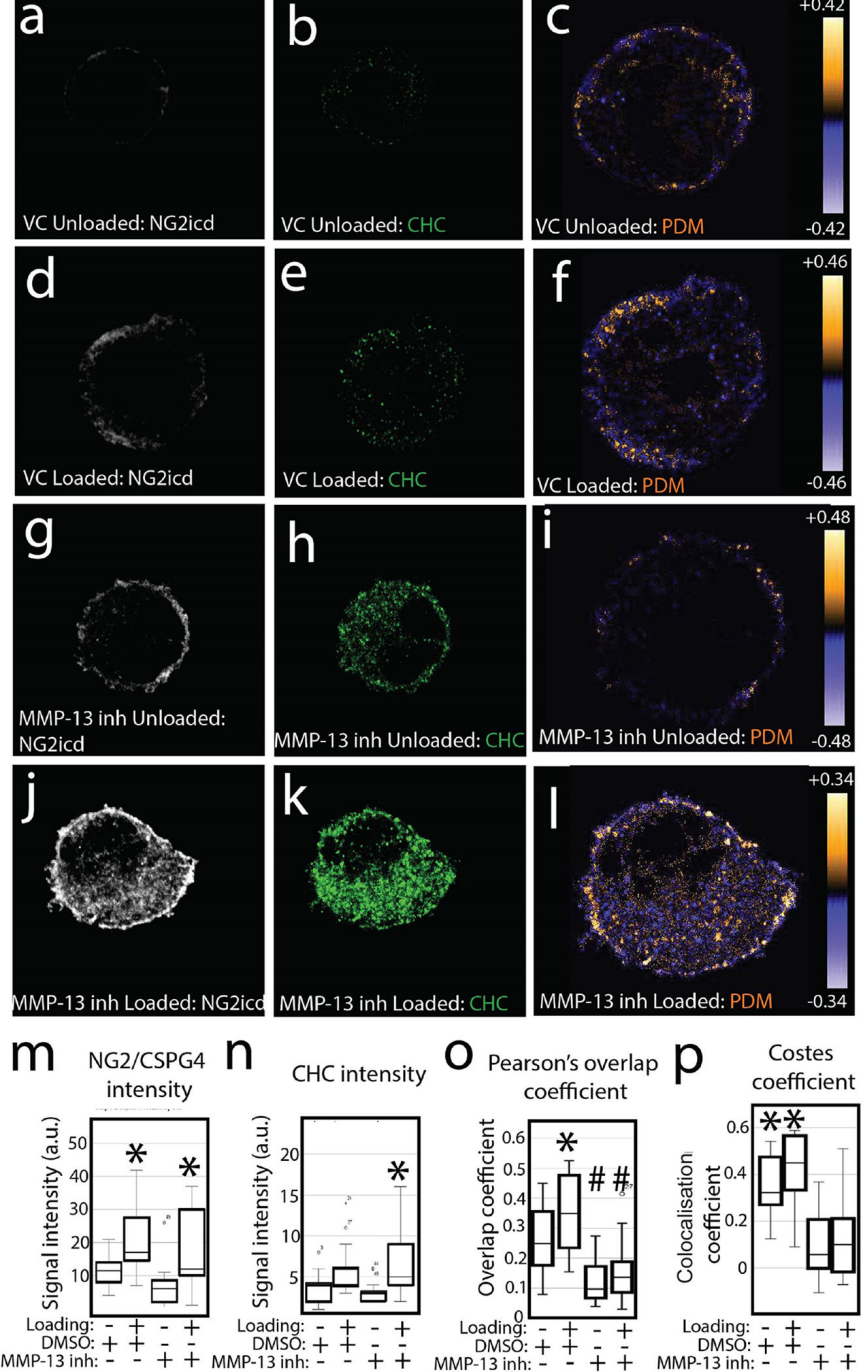
MMP-13 proteolysis potentiates the internalisation of the NG2/CSPG4 intracellular domain. (**a**,**b**) Immunocytochemistry for the NG2/CSPG4 intracellular domain (NG2icd); **a**) and CHC (**b**) in an unloaded primary mandibular fibrochondrocytes treated with DMSO. (**c**) PDM analysis illustrating overlap between the NG2icd and CHC with orange values representing positive overlap and blue colours representing negative overlap. (**d**,**e**) Immunocytochemistry for the NG2icd (**d**) and CHC (**e**) in a loaded primary mandibular fibrochondrocytes treated with DMSO, VC. (**f**) PDM analysis illustrating overlap between the NG2icd and CHC. (**g**,**h**) Immunocytochemistry for NG2icd (**g**) and CHC (**h**) in an unloaded primary mandibular fibrochondrocytes treated with an inhibitor for MMP-13 (MMP13inh). (**i**) PDM analysis illustrating overlap between the NG2icd and CHC. (**j**,**k**) Immunocytochemistry for the NG2icd (j) and CHC (**k**) in a loaded primary mandibular fibrochondrocytes treated with MMP13inh. (**l**) PDM analysis illustrating overlap between the NG2icd and CHC. (**m**) Quantification of intracellular NG2icd fluorescence intensity in DMSO and MMP13inh treated cells with and without loading. (**n**) Quantification of intracellular CHC fluorescence intensity inside the cell in DMSO (VC) and MMP13inh treated cells with and without loading. (**o**) Pearson’s overlap coefficient of intracellular NG2icd/CHC colocalisation in DMSO (VC) and MMP13inh treated cells with and without loading. (**p**) Costes method for intracellular NG2icd/CHC colocalisation bootstrapped against 25 random replicates. For Costes method, * = *p* > 0.95 for all samples. For all others, * = *p* < 0.05 for group comparisons between unloaded and loaded samples. # = *p* < 0.05 for group comparisons between DMSO (VC) and MMP-13 inhibited samples. *n* = 20/experimental group for quantification of the colocalisation analysis.

The release of the NG2/CSPG4 intracellular domain into the cytosol has been linked with TMJ OA and mechanical loading in mandibular fibrochondrocytes (Reed et al., 2022), potentially through regulated intramembrane proteolysis (Sakry et al., 2014; Sakry and Trotter, 2016). To determine if the internalisation of the NG2/CSPG4 intracellular domain is regulated by MMP-13 proteolytic activity, cell-agarose-collagen scaffolds were loaded and analysed by immunocytochemistry with a monoclonal antibody against the NG2/CSPG4 intracellular domain (NG2icd) and endocytosis marker CHC. Overlap visualisation using the PDM showed that mechanical loading resulted in high levels of NG2icd/CHC overlap in the cytosol of the DMSO treated samples (Fig. 4a–c *versus* 4d–f). There was also NG2icd/CHC overlap in the MMP-13 inhibited samples, but the overlap was restricted to the cell membrane after mechanical loading (Fig. 4g–i *versus* 4j–l). Colocalisation analysis showed that NG2/CSPG4 signal intensity was elevated after mechanical loading in both the DMSO-and MMP-13-inhibited samples (Fig. 4m) but CHC was only elevated in the MMP-13 inhibited sample (Fig. 4n). Pearson’s overlap coefficients between NG2icd/CHC within the cell were significantly elevated in the DMSO, but not MMP-13 inhibited, samples (Fig. 4o). Colocalisation, calculated by the Costes Coefficient, was found to be significant for all iterations in the DMSO samples (22/22 unloaded, 19/19 loaded; *p* > 0.95) but less than 50 % of the MMP-13 inhibited samples (7/14 unloaded, 3/14 loaded; *p* > 0.95; Fig. 4p). Together, these findings indicated that MMP-13 mediated cleavage of the NG2/CSPG4 ectodomain was important for release of the intracellular domain into the cytosol.

### Inhibition of MMP-13 altered OA and mineralisation genes in an NG2/CSPG4 and loading dependent manner

**Fig. 5.**
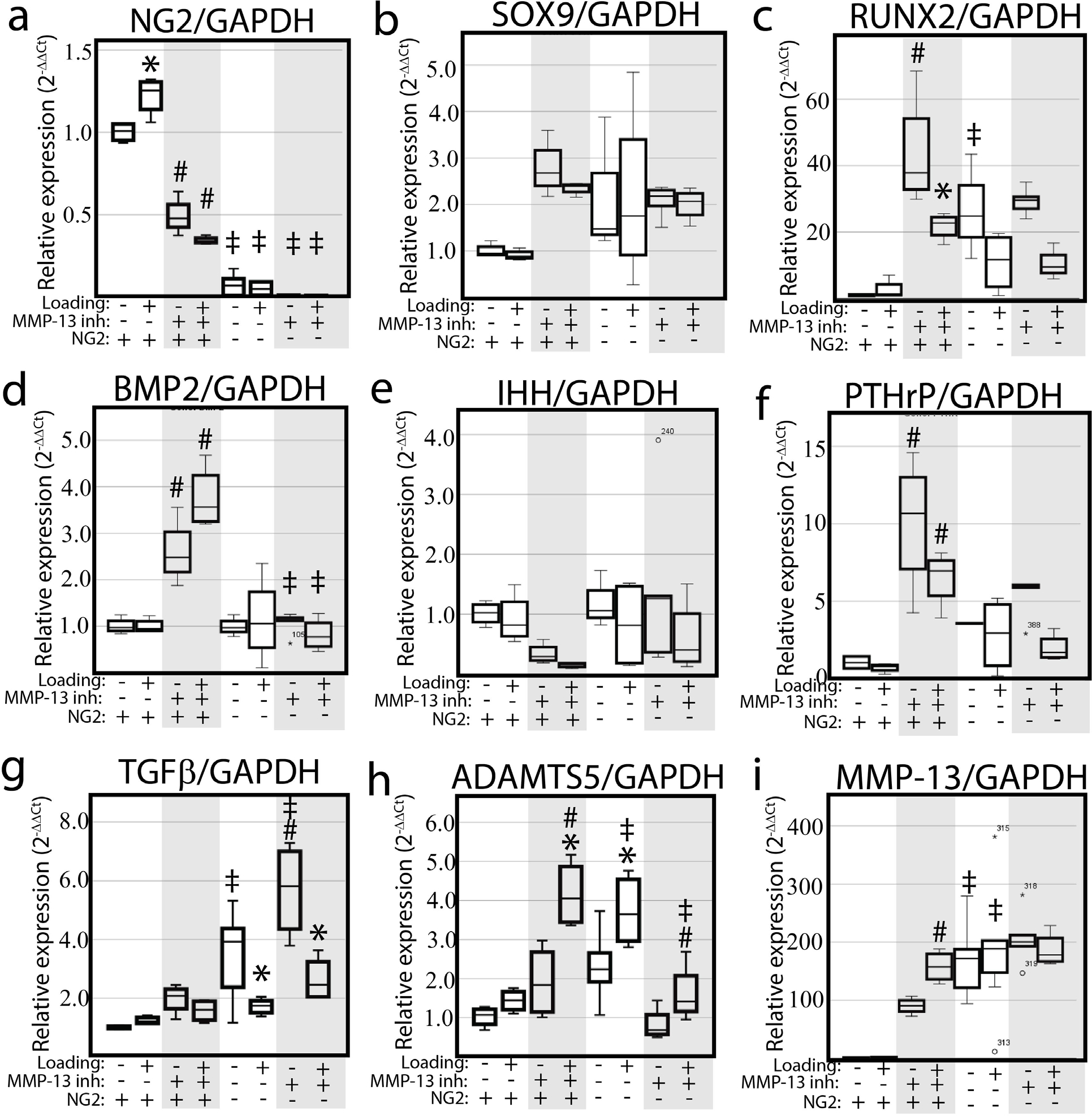
MMP-13 activity regulates key OA and mineralisation genes in a loading and NG2/CSPG4 dependent manner. (**a**,**b**,**c**,**d**,**e**,**f**) RT-qPCR from mRNA isolated from unloaded or loaded primary mandibular fibrochondrocytes treated with either DMSO or MMP13inh. Data standardised to the unloaded vehicle control sample and normalised to GAPDH using the ΔΔCq method. Gene expression of NG2/CSPG4 (**a**), Sox9 (**b**), Runx2 (**c**), BMP2 (**d**), IHH (**e**), PTHrP (**f**), TGFβ (**g**), ADAMTS5 (**h**), and MMP-13 (i). * = *p* < 0.05 for group comparisons between unloaded and loaded samples. # = *p* < 0.05 for group comparisons between DMSO and MMP-13 inhibited samples. ‡ = *p* < 0.05 for group comparison between control and NG2/CSPG4 knockout groups. *n* = 4 / genotype and experimental group.

Since hypertrophic mandibular fibrochondrocytes are positive for both MMP-13 and internalised NG2/CSPG4 and internalised NG2/CSPG4 is associated with mechanical loading, the expression of key mineralisation and OA genes in loaded and unloaded control and NG2/CSPG4 knockout cells treated with and without an inhibitor of MMP-13 were quantified. In control cells, inhibiting MMP-13 significantly suppressed NG2/CSPG4 and promoted Runx2, BMP2, PTHrP, and MMP-13 expression. Changes in PTHrP was associated with a non-significant reduction in IHH expression. These MMP-13 inhibition mediated changes in gene expression were rescued in NG2/CSPG4 knockout cells for Runx2, BMP2, and PTHrP. NG2/CSPG4 knockout cells had significantly higher expression of MMP-13, ADAMTS5, and TGFβ. Treatment of the NG2/CSPG4 knockout cells with the MMP13inh only rescued the expression of ADAMTS5. Mechanical loading suppressed the expression of Runx2 and TGFβ in MMP13inh treated control cells and in NG2/CSPG4 knockout cells, while ADAMTS5 was elevated in response to mechanical loading. Together, these expression data showed that key OA and mineralisation genes were altered in an MMP-13, NG2/CSPG4, and/or mechanical loading dependent manner, illustrating a complex mechanosensitive regulatory signalling axis (Fig. 5).

## Discussion

This study addressed the role of MMP-13 in proteolytic processing of NG2/CSPG4 when the mechanical homeostasis of the TMJ was disrupted. It was demonstrated that the reduction of full-length NG2/CSPG4 and internalisation of the intracellular domain was spatiotemporally correlated with MMP-13 immunopositive cells. It was also shown that MMP-13 regulated the retention of the NG2/CSPG4 in the extracellular matrix and that inhibiting MMP-13 led to the retention of NG2/CSPG4 on the cell membrane but did not affect the formation of mechanical-loading-dependent variant-specific fragments of the ectodomain. MMP-13 was also necessary for mechanical loading mediated internalisation of the NG2/CSPG4 intracellular domain. The MMP-13/NG2 signalling axis also regulated key biomineralisation and OA genes. Together, these findings mechanistically linked post-translational modification of NG2/CSPG4 with the mechanobiological regulation of growth and disease in the MCC.

Elevated MMP-13 protein was identified in both the superficial/articular layer cells and in the synovial fluid, illustrating that this protease was present during a time and location that could affect NG2/CSPG4 processing during the progression of TMJ OA. Surprisingly, MMP-13 protein was also recovered from the synovial fluid CL to the discectomy. This finding was consistent with reports that degenerative changes occur CL to the procedural side, using the unilateral partial discectomy preclinical model of TMJ OA (Cohen et al., 2014; Yotsuya et al., 2019) and could reflect a need to limit condylar displacement and/or joint reaction forces on the CL side after injury (Yotsuya et al., 2020). A similar pattern was identified from the clinical samples taken from patients with unilateral degenerative TMJ OA. The presence of synovial MMP-13 CL to the treatment side could indicate a subclinical stage of degeneration, an underlying chronic inflammatory condition, or overloading on the non-injury side due to compensatory jaw mechanics. This observation indicated that MMP-13 may not be a reliable biomarker of acute TMJ arthropathy requiring urgent clinical intervention.

MMP-13 is a sheddase of NG2/CSPG4, possibly processing the shed, membrane tethered fragment (Asher et al., 2005; Joo et al., 2014). During TMJ OA, there is a loss of the shed-membrane tethered NG2/CSPG4 fragments. This fragment consists of the ectodomain retained near the cell membrane, but lacking the intracellular domain (Reed et al., 2022). Inhibiting MMP-13 in unstimulated primary fibrochondrocytes did not change the amount of full length, shed-membrane tether, or media associated shed NG2/CSPG4 fragments in monolayer cell culture when compared with a vehicle control. This finding was in contrast to anoikis-induced MMP-13 activation in periodontal fibroblasts, with inhibition of MMP-13 decreasing the levels of media-associated shed NG2/CSPG4 (Joo et al., 2014). The current study indicated that basal level proteolytic cleavage of the NG2/CSPG4 ectodomain was either not significantly affected by MMP-13 activity or undetectable due to sequestration of cleaved fragments on the membrane or in the matrix. Immunostaining of decellularised matrix in both the on-cell western blot and immunocytochemistry studies illustrated that MMP-13 was important for the retention of NG2/CSPG4 pericellular matrix. Since the NG2/CSPG4 ectodomain binds with type VI collagen (Burg et al., 1996; Tillet et al., 2002) and since primary mandibular fibrochondrocytes produce high levels of endogenous type VI collagen (Reed, *unpublished*), it is hypothesised that MMP-13 acts in a localised fashion under basal level conditions to regulate cell-matrix mediated signalling networks. This hypothesis is consistent with previous findings by the authors in TMJ OA (Yotsuya et al., 2019), with other reports on NG2-type VI collagen interactions (Cattaruzza et al., 2013; Sardone et al., 2016; Sardone et al., 2014), and with MMP-13 mediated processing of NG2/CSPG4 during anoikis (Joo et al., 2008).

To characterise the role of MMP-13 under activated conditions, an *in vitro* mechanical loading model was used. This *in vitro* model is germane to studies of TMJ OA since mechanical overloading or disruptions in mechanical homeostasis are key initiating conditions for degenerative arthropathy (Tanaka et al., 2008). Further, since the experimental design of the current study used agarose-collagen embedded cells, the compression bioreactor was a closed system similar to the *in vivo* condition *(i.e.* shed protein and proteases cannot diffuse or be released through the media). It was shown that constrained static compression resulted in no significant increase in the total MMP-13 but a significant increase in the active form of MMP-13. The activation of latent MMP-13 can occur through both MMP2 and/or MMP14 (Knäuper et al., 1996). MMP14 is of interest as it is a protease that cleaves the distal most N-terminal region of NG2/CSPG4 and is implicated in cell repair/regeneration (Nishihara et al., 2015). MMP-2 is also associated with the progression of OA (Zeng et al., 2015), is implicated in NG2/CSPG4 functionality (Iida et al., 2007), and could be therapeutically targeted with copper-methionine (Bagheri Varzaneh et al., 2016; Varzaneh et al., 2017). Together, MMP14 and/or MMP-2 could potentially modify the NG2/CSPG4 ectodomain and simultaneously activate MMP-13, leading to subsequent cleavage of the NG2/CSPG4 ectodomain closer to the transmembrane region of the protein.

Mechanical loading was also associated with a significant decrease in full length and a non-significant decrease in the shed, membrane-tethered fragment of NG2/CSGP4, matching the pattern measured *in vivo* (Reed et al., 2022). When MMP-13 was inhibited, unloaded cells were associated with a non-significant increase in full length NG2/CSGP4 and a significant increase in the shed, membrane-tethered fragment. Together, these data illustrate that mechanical activation of MMP-13 is involved in processing of the NG2/CSPG4 ectodomain and leads to the accumulation of the shed, membrane-tethered fragment. However, mechanically activated MMP-13 is not the only protease that is responsible for the cleavage of the shed-membrane tethered fragment. Here the current study focused on the 300 kDa full-length fragment and the 275/260 kDa shed, membrane tethered fragment, but the data suggested at least three additional lower molecular weight bands. This pattern of proteolytic processing was consistent with MMP9, also yielding five separate NG2/CSPG4 fragments between 200–80 kDa (Larsen et al., 2003). MMP9 is a gelatinase that regulates the cellular response to inflammation, is correlated with lysyl oxidase, and is stimulated by mechanical loading (Honda et al., 2000). Additional mechanistic studies are needed to define select and specific functionality of these two proteases, their sequence of activation, and their precise role in clearing NG2/CSPG4 from cells following injury, including the identification of specific proteases that generate the multiple fragments associated with sequential processing of the ectodomain.

In other cell types, the proteolytic processing of the NG2/CSPG4 ectodomain is associated with the internalisation of the intracellular domain through regulated intramembrane proteolysis (Sakry et al., 2014; Sakry and Trotter, 2016) and clathrin mediated endocytic processing (Feutlinske et al., 2015; Reed et al., 2022; Yotsuya et al., 2019). The current study illustrated that mechanical loading could induce the internalisation of the NG2/CSPG4 intracellular domain, with NG2/CSPG-CHC overlap coefficients significantly increasing after mechanical loading and significant colocalisation using the Costes bootstrap method. The function and fate of NG2/CSPG4 after internalisation has yet to be fully resolved. This subcellular localisation pattern could indicate degradation through the late endosome/lysosome machinery or it could indicate cell surface recycling or repositioning on the cell in response to external biophysical stimuli (Daynac et al., 2018). There is precedent for NG2/CSPG4 cell surface recycling during adhesion and motility dynamics (Feutlinske et al., 2015; Makagiansar et al., 2004). When MMP-13 is inhibited, the overlap of NG2/CSPG4 with CHC in the cytosol is significantly reduced, and these two proteins fail to colocalise within the cell. Since MMP-13-mediated proteolysis of the NG2/CSPG4 ectodomain is associated with retention of the ectodomain in the extracellular matrix, this specific proteolytic step is predicted to precede the formation of clathrin-coated pits containing the intracellular domain during endocytic transport.

NG2/CSPG4 functionality has been contextually linked with biomineralisation in cartilages. High levels of NG2/CSPG4 internalisation are observed in both hypertrophic chondrocytes and in superficial/articular layer cells during TMJ OA (Reed et al., 2022; Yotsuya et al., 2019), NG2/CSPG4 protein is present in growth plate cartilage and in cranial sutures during growth (Fukushi et al., 2003; Nishiyama et al., 1991), and loss of the type VI collagen extracellular matrix ligand impairs endochondral ossification in the TMJ and alveolar bone (Komori et al., 2022a; Komori et al., 2022b). The current study mechanistically linked NG2/CSGP4 with key biomineralisation and OA genes in mandibular fibrochondrocytes, illustrating MMP-13, NG2/CSPG4, and mechanical loading dependent expression of PTHrP and BMP2. NG2/CSPG4-dependent increases in TGFβ, Runx2, and MMP-13, and mechanical-loading dependent suppression of RUNX2 and TGFβ were also found. The high levels of MMP-13 in NG2/CSPG4 knockout cells supports the hypothesis of a co-regulatory feedback loop proposed by Jamil et al. (2016) and implicates NG2/CSPG4 in other studies linking MMP-13 activation with biomineralisation pathways (Aref-Eshghi et al., 2015; Jahangir et al., 2020).

Limitations of this study included not characterising the role of the chondroitin sulphate chains in NG2/CSPG4 functionality. For western-blot characterisation, the chondroitin sulphate chains are removed. These chains could participate in the sequestration of growth factors or retention of the ectodomain on the membrane or matrix. Separate but parallel proteolytic processing of the chondroitin sulphate chains is a potential confounding variable in the study. A second limitation of the study was that NG2/CSPG4 can be released from cells in exosomes, with a predicted molecular weight between 250–290 kDa (Christianson et al., 2013; Tamburini et al., 2019). For characterisation of NG2/CSPG4 in decellularised samples, the ectodomain could be present in the matrix in exosomes and not retained following cleavage. A third limitation was that *in vitro* data were collected from healthy primary cells from young mice. This age was chosen following published protocols indicating that the cells maintain their chondrogenic phenotype (Bougault et al., 2009; Gosset et al., 2008). However, it is acknowledged that aged or OA primary cells may be transcriptionally distinct from the primary cells used in this study, altering the expression and processing of NG2/CSPG4.

To summarise, the current study illustrated that NG2/CSPG4 proteolysis by MMP-13 generated variant-specific functions that could potentiate complex and multifaceted inside-out and outside-in signal transduction. These variant specific functions in response to dysfunctional proteolysis of the ectodomain can result in the accumulation of membrane associated NG2/CSPG4 on the cell, potentially affecting viability (Joo et al., 2008; Joo et al., 2014), inhibiting cell migration by inferring with binding to the extracellular matrix (Tang et al., 2018), inhibiting cell maturation and differentiation (Larsen et al., 2003), and/or impacting ligand-receptor binding by controlling the concentration and presentation of growth factors such as FGF2 and PDGF (Goretzki et al., 1999). The role of MMP-13 in regulating NG2/CSPG4-FGF2 interactions could be particularly informative in the study of OA pathophysiology. In the appendicular skeleton, FGF2 is a mitogenic growth factor that is released from perlecan in the pericellular matrix during mechanical loading and during OA progression (Vincent et al., 2007) and activates RUNX2 to regulate the expression of MMP-13 through ERK 1/2 (Im et al., 2007; Raggatt et al., 2006; Wang et al., 2004). Thus, NG2/CSPG4 is in a privileged subcellular location to integrate and transduce MMP-13 mediated cross talk between cell signalling networks and growth factors in response to the pathogenic changes in the pericellular matrix occurring during the progression of OA in both primary and secondary cartilages.

## Figures and Tables

**Table 1:** Primer sequences for real-time quantitative reverse transcriptase polymerase chain reaction (qRT-PCR)

	Forward	Reverse

**NG2/CSPG4**	TCCTTCTTCGGGGAGAACCA	TGGTCATCTTGGCCTGCTGC
**SOX9**	AATGCTATCTTCAAGGCGCTG	GGACCCTGAGATTGCCCAG
**RUNX2**	TGTTCTCTGATCGCCTCAGTG	CCTGGGATCTGTAATCTGACTCT
**BMP2**	TGAGCAAAGTGCTTGCACAC	AGCCCCCTGGAAGGGATTAT
**IHH**	TGCTGGCGCGCTTAGCAGTG	GCAGCGGCCGAATGCTCAGA
**PTHrP**	ATTCCTACACAAGTCCCAGAG	ACTTGCCCTTGTCATGCAGTA
**TGFβ1**	CACCTGCAAGACCATCGACAT	GAGCCTTAGTTTGGACAGGATCTG
**ADAMTS5**	CTGCCCACCCAATGGTAAGT	GTAATTTCTGCCCAGCGTGC
**MMP13**	TGATGGACCTTCTGGTCTTCTGG	CATCCACATGGTTGGGAAGTTCT

## Data Availability

All relevant data are within the paper.

## List of Abbreviations

AD: articular disc
ADAM10: a disintegrin and metalloproteinase domain-containing protein 10
ADAMTS5: a disintegrin and metalloproteinase with thrombospondin motifs 5
AN2: Antje Niehaus 2
ANOVA: analysis of variance
a.u.: arbitrary units
BMP2: bone morphogenetic protein 2
cDNA: complementary DNA
CHC: clathrin heavy chain
CL: contralateral
CSPG4: chondroitin sulphate proteoglycan 4
DAPI: 4′,6-diamidino-2-phenylindole
DDR2: discoidin domain receptor 2
DMEM: Dulbecco’s modified Eagle medium
DMSO: dimethylsulphoxide
EDTA: ethylenediaminetetraacetic acid
ERK1/2: extracellular signal-regulated kinase1/2
FBS: foetal bovine serum
FGF2: fibroblast growth factor 2
GAPDH: glyceraldehyde-3-phosphate dehydrogenase
HTRA1: high temperature requirement A serine peptidase 1
IHH: Indian hedgehog protein
IL: ipsilateral
MCC: mandibular condylar cartilage
MMP: matrix metalloproteinase
MMP13inh: MMP-13 inhibitor
NG2: neuron glial antigen 2
NG2icd: neuron glial antigen 2 intracellular domain
NSC: non-surgical control
OA: osteoarthritis
OCT: optimal cutting temperature
PBS: phosphate-buffered saline
PDGF: platelet-derived growth factor
PDM: product of the difference from the mean
PFA: paraformaldehyde
PKCα: protein kinase C alpha
PTHrP: parathyroid hormone-related protein
RT-qPCR: quantitative reverse transcription polymerase chain reaction
Runx2: Runt-related transcription factor 2
TBS: tris-buffered saline
TGFβ: transforming growth factor beta
TIMP: tissue inhibitor of metalloproteinases
TMJ: temporomandibular joint
UIC: University of Illinois Chicago
VC: vehicle control
